# The Weekend Effect on Urban Bat Activity Suggests Fine Scale Human-Induced Bat Movements

**DOI:** 10.3390/ani10091636

**Published:** 2020-09-11

**Authors:** Han Li, Chase Crihfield, Yashi Feng, Gabriella Gaje, Elissa Guzman, Talia Heckman, Anna Mellis, Lauren Moore, Nayma Romo Bechara, Sydney Sanchez, Samantha Whittington, Joseph Gazing Wolf, Reuben Garshong, Kristina Morales, Radmila Petric, Lindsey A. Zarecky, Malcolm D. Schug

**Affiliations:** 1 Department of Biology, University of North Carolina Greensboro, Greensboro, NC 27412, USA; chase.a.crihfield@gmail.com (C.C.); yashifeng@gmail.com (Y.F.); gmsgaje@gmail.com (G.G.); elisssaguzman@gmail.com (E.G.); tbheckma@gmail.com (T.H.); annam.mellis@gmail.com (A.M.); laurenmoore1967@gmail.com (L.M.); naymaromo@gmail.com (N.R.B.); sanchezsydney94@gmail.com (S.S.); sammiw822@gmail.com (S.W.); shunkaha3@gmail.com (J.G.W.); rngarsho@uncg.edu (R.G.); KristinaCMorales@gmail.com (K.M.); r_petric@uncg.edu (R.P.); mdschug@uncg.edu (M.D.S.); 2 School of Life Sciences, Arizona State University, Tempe, AZ 85281, USA; 3 Greensboro Science Center, Greensboro, NC 27455, USA; lzarecky@greensboroscience.org

**Keywords:** weekend effect, bats, urban ecology, disturbance, movements, city parks, greenways

## Abstract

**Simple Summary:**

On weekends, people do things differently from weekdays, such as dining at a restaurant, going to a night club, attending a concert or a sporting event, or simply staying up late. These leisure activities in the city can change the environment people live in and can hurt wildlife that also lives in the same city. We recorded bats in the city center and in the city periphery and compared how active bats were. We found that in the city center, bats were less active on weekends than weekdays. The opposite pattern was found in the city periphery. It is possible that bats moved from the city center to the city periphery on weekends. Thus, continuous greenways are important to facilitate bat movements and avoid human–wildlife conflict. City planners can add new parks and/or preserve old-growth vegetation to form the center-to-periphery greenways.

**Abstract:**

In the urban environment, wildlife faces novel human disturbances in unique temporal patterns. The weekend effect describes that human activities on weekends trigger changes in the environment and impact wildlife negatively. Reduced occurrence, altered behaviors, and/or reduced fitness have been found in birds, ungulates, and meso-carnivores due to the weekend effect. We aimed to investigate if urban bat activity would differ on weekends from weekdays. We analyzed year-round bat acoustic monitoring data collected from two sites near the city center and two sites in the residential area/park complex in the city periphery. We constructed generalized linear models and found that bat activity was significantly lower on weekends as compared to weekdays during spring and summer at the site in the open space near the city center. In contrast, during the same seasons, the sites in the city periphery showed increased bat activity on weekends. Hourly bat activity overnight suggested that bats might move from the city center to the periphery on weekends. We demonstrated the behavioral adaptability in urban wildlife for co-existing with human. We recommend that urban planning should implement practices such as adding new greenspaces and/or preserving old-growth vegetation to form continuous greenways from the city center to the city periphery as corridors to facilitate bat movements and reduce possible human-wildlife conflict.

## 1. Introduction

More than half of the world’s people live in urban areas currently [1]. It is estimated that urban land cover may triple from 2000 to 2030 globally [2]. In the United States, urbanization is one of the main drivers of land cover and land use change and it is anticipated to have an expanding rate in the future [3,4]. Urban areas are designed and constructed to meet human needs [5]. However, studies demonstrate that wildlife is inhabiting urban areas and adapting to urbanization in a process called synurbanization [6].

During synurbanization, wildlife faces novelties of urban ecosystems that would not be encountered in wildlands. One of the novelties is human disturbances with increased intensity or unique temporal patterns [7,8]. In urban areas human disturbances can be the presence of human (with or without direct interaction with wildlife) or environmental changes caused by human activities [9,10]. Wildlife species that are successful in synurbanization usually have a high tolerance to human disturbances by changing their behaviors [6]. Studies documented behavioral changes reflecting synurbanization such as increased aggression, boldness, and vigilance towards humans and other animals in mammals (e.g., [11,12,13,14]) and reduced singing in response to ambient noise in birds (e.g., [15,16]). 

Temporal patterns of human disturbances may vary considerably and thus impact animal behavior and adaptation to urban environments. One well established example of temporal cycling in human disturbances is the weekend effect. Meteorologists demonstrated that increases in leisure activity on weekends exert the release of aerosol pollutants to the atmosphere in urban areas triggering seasonally and geographically specific changes in precipitation and temperature (e.g., [17,18,19,20,21]). Elevations in ambient noise and night-time illumination have also been documented on weekends as compared to weekdays (e.g., [22,23]).

Wildlife may alter their behaviors to adapt to increased human activity on weekends and related temporal environmental changes. It has been documented that leisure activities in recreational areas occur at a higher frequency on the weekends than during weekdays and thus exert more disturbances on both diurnal and nocturnal species over the weekends [24,25,26]. The weekend effect of human presence on wildlife is often negative, resulting in reduced numbers of active individuals, increased avoidance to areas of human presence, altered behaviors, even reduced fitness [27,28,29]. Evidence also suggests elevated noise and nighttime illumination might affect wildlife negatively in a way similar to human presence [30,31,32].

Bats inhabit urban areas worldwide. Certain bat species are better at adapting to the urban environment than others [33,34,35]. Nocturnal insectivorous bat activity peaks within the first few hours following sunset [36,37], which is a pattern that overlays temporally with human leisure nightlife. In the current literature, there is no evidence suggesting that urban bats avoid flying over human crowds or areas of high human activity. However, human presence in or around bat roosts can negatively impact bats resulting in delayed emergence, reduced activity, and roost abandonment [38,39,40]. The impact of environmental stressors such as elevated noise, nighttime illumination, and traffic associated with leisure nightlife may also reduce bat activity [41,42,43,44].

Our study aimed to investigate the weekend effect on local weather and urban bat activity and to extrapolate which factor, weather conditions or human presence would primarily influence the weekend effect as evidenced through bat activity within a city (Appendix A). As bats are nocturnal, we consider Friday and Saturday nights as weekends and the rest as weekdays. We hypothesized that the weekend effect would be observed in abiotic weather variables. As our study was conducted in the southeast United States, we predicted that weekends would have higher temperature and lower precipitation than weekdays [19,20]. We also hypothesized that bat activity, both overall and species-specific, would be the same between weekends and weekdays. This is because bats would avoid areas where more people enjoyed leisure nightlife on weekends, whereas the altered weather conditions (higher temperature and lower precipitation) on weekends could favor bats to be active in our study area [37]. 

## 2. Materials and Methods 

### 2.1. Study Sites and Bat Acoustic Monitoring Design

We selected four bat monitoring sites at two locations in Greensboro, North Carolina, USA. Greensboro is a medium-sized city in the Piedmont region with a population near 300,000. The climate of Greensboro is temperate/humid subtropical in the Koppen climate classification, featuring four distinct seasons [45]. Previous studies documented the following bat species in Greensboro: big brown bat (*Eptesicus fuscus*), eastern red bat (*Lasiurus borealis*), hoary bat (*Lasiurus cinereus*), silver haired bat (*Lasionycteris noctivagans*), evening bat (*Nycticeius humeralis*), tricolored bat (*Perimyotis subflavus*), and Mexican free-tailed bat (*Tadarida brasiliensis*) [37,46]. All sites in this study are part of a long-term urban wildlife monitoring effort in Greensboro.

The first pair of bat monitoring sites are located at the Greensboro Science Center (GSC), which includes an aquarium and a zoological park accredited by the Association of Zoos and Aquariums. The GSC sits within a 2.2 km^2^ wooded area comprised of a federal park, a city park and the GSC campus. The park complex is located approximately 7.5 km northwest of the downtown city center. The lands surrounding the park complex are predominantly residential neighborhoods, featuring single family houses with scattered commercial and office zones according to the Greensboro Planning Department (Figure S1). The first site at GSC is in an open area on the rooftop of a single story building (no closed tree canopy within 50 m diameter, named as GSC open). The second site is in the adjacent forest (closed tree canopy with a 50 m diameter, named as GSC woods). 

The second pair of bat monitoring sites are located on the University of North Carolina Greensboro (UNCG) campus. The UNCG campus is adjacent to Downtown Greensboro and surrounded by commercial use lands within the Greensboro central business zone (Figure S1). The two bat monitoring sites on the UNCG campus are physically comparable to the two sites at GSC. The first site at UNCG is an open grassland area with scattered trees (50 m in diameter of the opening, trees do not form a closed canopy, named as UNCG open). The second site at UNCG is within the Peabody park forest (closed tree canopy, named as UNCG woods). Both sites at UNCG were used as control sites for an urban wetland construction project (see detailed descriptions of vegetation and physical conditions of both sites in Parker et al. [47]).

At each site, we used a Song Meter SM4BAT-FS ultrasonic recorder and a SMM-U2 microphone (Wildlife Acoustics Inc., Maynard, Massachusetts, USA) to monitor bat activity. The SM4BAT-FS recorder was secured to a tree or the ground. The microphone was extended via cables and PVC pipes to approximately 8 m above the ground, except for the GSC open detector, which was 15 m above the ground (placed on the rooftop of a single story building for equipment security and public safety reasons). At the GSC open site, PVC pipes were held straight up by a weigh station (Figure S2). At the three other sites, PVC pipes were strapped to a tree and another 1 m PVC pipe was placed parallel to the ground to position the microphone away from the tree trunk. All recorders were set to record bat acoustic activities from sunset to sunrise nightly, continuously throughout the year. The specific recorder settings and power sources are described in Parker et al. [47] and Springall et al. [48]. All recorders were maintained by UNCG or GSC designated personnel every two to four weeks and recordings were downloaded to an external hard drive during each maintenance. All fieldwork followed the American Society of Mammalogist’s guidelines for use of wild animals in research and was approved by the North Carolina Wildlife Resources Commission (permit number 17-SC00162, 18-SC00162, 19-SC00162).

### 2.2. Extraction of Bat and Weather Variables 

We collected bat recordings from March to November in 2018 and 2019 (a total of 73 calendar weeks). We used Kaleidoscope (version 4.4, Wildlife Acoustics Inc., Maynard, Massachusetts, USA) to automatically process recordings and identify recordings to species. We defined a bat pass as a recording that included at least three complete bat echolocation call pulses within 0.5 s. To identify a bat pass to the species level, we used Kaleidoscope automatic identification with the accuracy set to neutral. We selected species described in the introduction as the candidate species. Furthermore, we accepted the species identification only when a bat pass had a match ratio greater than 0.60, which is a criterion for accurate species identifications generated by comparing automatic and manual identification results in the study area (e.g., [37,47,49]). All other bat passes were classified as “no ID”. 

We summarized total bat activity and species-specific bat activity from the identification results after correcting for night length. The night length varies between months in Greensboro, therefore, we used R package “suncalc” [50] to extract night length for each recording night. Using night length, we standardized total bat activity (all species including no ID) and species-specific bat activity as number of bat passes per recording hour as used by previous similar studies (e.g., [51,52]). We downloaded weather data from the weatherSTEM database for UNCG (https://guilford.weatherstem.com/data?refer=/uncg) between March and November in 2018 and 2019. We collected daily mean temperature (°C), daily mean wind speed (km/h), and daily total precipitation (cm) for each recording night. All sites had the same weather data. We used meteorological seasons: spring (March–May), summer (June–August), and fall (September–November) to assign data to a season and conducted all statistical analyses season by season.

### 2.3. Statistical Analysis 

We conducted all statistical analyses and data visualization in R (version 3.6.3, [53]). We used an α level of 0.05 for statistical significance criterion for all analyses. We tested dependent variable normality with the Shapiro–Wilk test and specified when we used distributions other than a Gaussian distribution [54]. Since the independent variable was the day of the week, we first excluded weeks of incomplete data (bat detectors malfunctioned, no weather data, or weeks overlaid with two seasons). It is important to note that the recorded bat activity naturally varied among sites because of differences in obstacles within the recording acoustic space such as canopy cover or sound transmission interference [55,56]. Therefore, we treated each site separately in the statistical analyses. 

To evaluate if the weekend effect was evident in the weather data, we constructed generalized linear models for each weather variable versus day of the week with Monday as the reference level. To incorporate possible variation between years, we included the categorical covariate year with year 2018 as the reference level. The generalized linear model for daily precipitation was fitted with a quasi-Poisson distribution. We plotted the model residuals versus fitted values to visually examine the fit of each model [54]. We also constructed similar generalized linear models to evaluate if the weekend effect was evident in total and species-specific bat activity. We included weather variables that did not exhibit the weekend effect and year as covariates. These covariates were dropped if they were not significant in the corresponding models (indicated as “NA” in result tables). We fitted all bat variable models with a quasi-Poisson distribution. For the species-specific bat activity, we only conducted the analysis for the summer season at the two open sites (GSC open and UNCG open) because bat activity was too low for other sites/seasons. If we found a significant weekend effect on total bat activity, we also plotted hourly total bat passes by the day of the week (Friday, Saturday, and weekday average) to visualize how bat activity changed over a night. All model structures were listed in Table S3.

## 3. Results

The mean daily temperature in Greensboro in 2018 and 2019 was 16.0 ± 7.1 °C, 25.6 ± 2.5 °C, and 17.0 ± 7.7 °C respectively for spring, summer, and fall. The mean daily wind speed in Greensboro in 2018 and 2019 was 6.9 ± 2.8 km/h, 5.0 ± 2.0 km/h and 4.8 ± 3.0 km/h respectively for spring, summer, and fall. The mean daily precipitation in Greensboro in 2018 and 2019 was 0.14 ± 0.04 cm, 0.19 ± 0.05 cm and 0.19 ± 0.05 cm respectively for spring, summer, and fall. The generalized linear models showed that there was no difference in weather measurements between days of the week and weather measurements were not different between 2018 and 2019 within each season (all *p* > 0.05, Table S3).

In 2018 and 2019, we collected 42 full weeks of recordings at the GSC open site and 38 full weeks of recordings at the GSC woods site, yielding 47,478 and 13,524 bat passes respectively. At the UNCG open site, we collected 64 full weeks of recordings with 106,485 bat passes. At the UNCG woods site, we collected 58 full weeks of recordings with 13,227 bat passes. Summer had the highest number of bat passes at all sites except for the UNCG woods site, where spring had the highest number of bat passes (Table 1 and Table 2).

**Figure 1:**
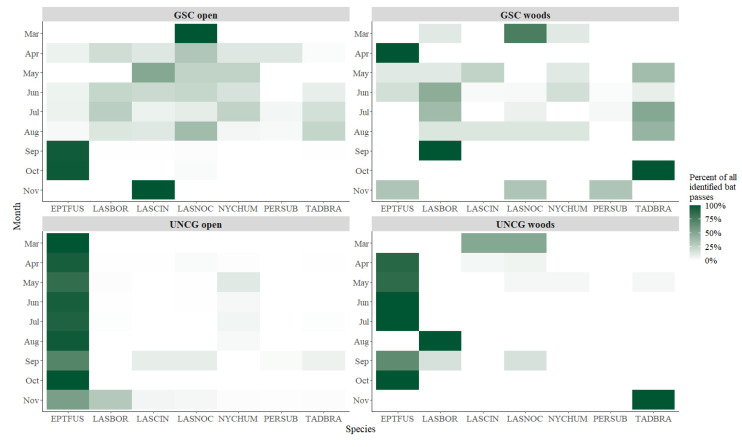
Monthly species-specific bat pass percentage at four sites (Greensboro Science Center/GSC and University of North Carolina Greensboro/UNCG) between 2018 and 2019 in Greensboro, North Carolina, USA.

In total, 105,768 bat passes (58.5% of all bat passes) met the identification criteria and were identified to species. At both UNCG sites, for most months the big brown bat was the most common species (Figure 1).

**Figure 2:**
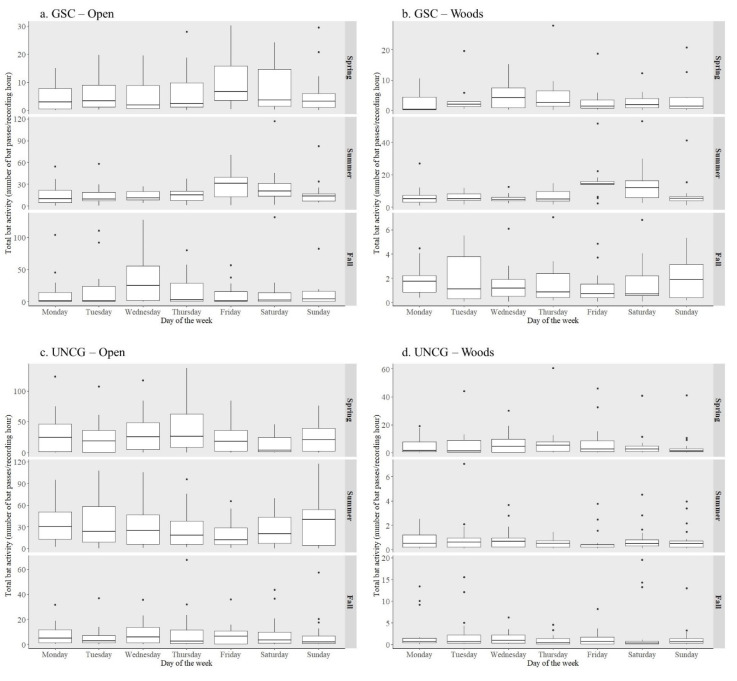
Quartile box plot of hourly total bat activity (all species bat passes combined) in relation to the day of the week by season from recordings collected in 2018 and 2019 at (**a**) the Greensboro Science Center (GSC) open site, (**b**) the Greensboro Science Center woods site, (**c**) the University of North Carolina Greensboro (UNCG) open site, (**d**) the University of North Carolina Greensboro woods site in Greensboro, North Carolina, USA.

**Figure 3:**
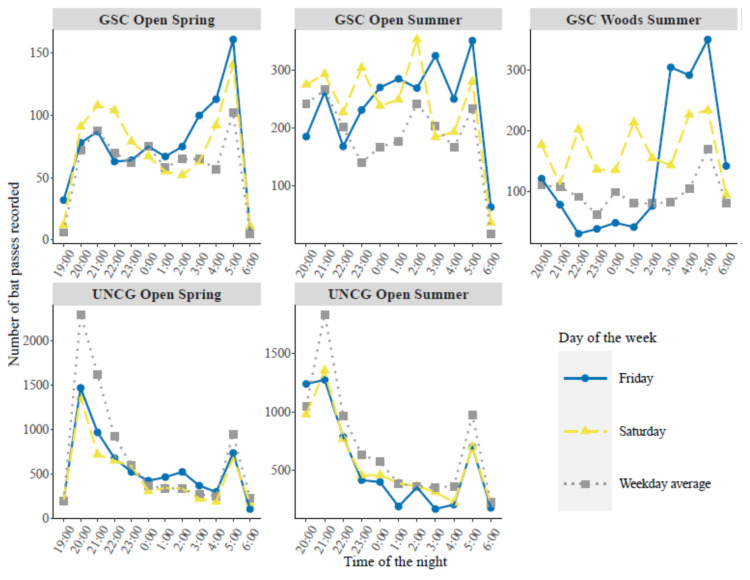
Hourly total bat activity plot to compare Friday, Saturday, and weekday average for sites and season where total bat activity was different on weekends from weekdays in Greensboro, North Carolina, USA. University of North Carolina Greensboro (UNCG); Greensboro Science Center (GSC).

At the GSC open site, the generalized linear model showed that total bat activity was significantly higher on Friday and Saturday nights in spring and summer (spring Friday regression estimate 0.611 ± 0.230, *p* = 0.010, spring Saturday regression estimate 0.496 ± 0.233, *p* = 0.036, summer Friday regression estimate 0.763 ± 0.293, *p* = 0.011, summer Saturday regression estimate 0.723 ± 0.292, *p* = 0.015); whereas other days of the week showed no difference with Monday as reference (Table 1). In spring, we recorded 54% more bat passes on Friday nights and 23% more on Saturday nights than on Monday nights; in summer we recorded 99% more bat passes on Friday nights and 73% more on Saturday nights than on Monday nights (Figure 2a). In both spring and summer, the hourly total bat activity plot showed that on Friday night, the increase occurred mostly after midnight as compared to the weekday average. On Saturday nights, total bat activity was generally higher than the weekday average throughout the night (Figure 3).

Similarly, at the GSC woods site total bat activity was significantly higher on Friday and Saturday nights in summer (Friday regression estimate 0.929 ± 0.230, *p* < 0.001, Saturday regression estimate 0.869 ± 0.231, *p* < 0.001, Table 1). In summer we recorded 135% more bat passes on Friday nights and 103% more on Saturday nights than on Monday nights at this site (Figure 2b). Hourly total bat activity plot showed a pattern similar to the GSC open site as well. The increase of total bat activity on Friday night occurred during the last few hours of the night, whereas on Saturday night total bat activity was higher than the weekday average throughout the night (Figure 3).

In contrast to sites at GSC, we found a significant decrease of total bat activity at the UNCG open site on Friday and Saturday nights in spring and summer (spring Friday regression estimate −0.321 ± 0.218, *p* = 0.043, spring Saturday regression estimate –0.660 ± 0.217, *p* = 0.016, summer Friday regression estimate –0.524 ± 0.257, *p* = 0.016, summer Saturday regression estimate –0.264 ± 0.232, *p* = 0.020, Table 2). In spring, we recorded 24% fewer bat passes on Friday nights and 55% fewer on Saturday nights than on Monday nights; in summer, we recorded 43% fewer bat passes on Friday nights and 23% fewer on Saturday nights than on Monday nights (Figure 2c). The hourly total bat activity plot showed that the decrease of bat activity occurred throughout the night for both spring and summer (Figure 3). There was no effect of day of the week on total bat activity at the UNCG woods site (Table 2, Figure 2d).

Species-specific generalized linear regression models showed that some species’ activities were affected by the day of the week (Table 3 and Table 4). At the GSC open site where summer total bat activity increased on weekends, we found a 57% increase in activity of the big brown bat on Saturday nights as compared to Monday; a 38% increase on Saturday and a 23% increase on Sunday nights for the red bat; a 45% increase on Friday and a 25% increase on Saturday nights for the silver hair bat; and a 61% increase on Friday nights for the evening bat (Table 3). At the UNCG open site where summer total bat activity decreased on weekends, we found the decrease of activity for three species on both Friday and Saturday nights (Table 4). As compared to Monday, Friday nights bat activity decreased 22%, 38%, and 19% for the big brown bat, the red bat, and the evening bat, respectively. On Saturday nights, the bat activity decreased 16%, 22%, and 24% for the big brown bat, the red bat, and the evening bat, respectively.

## 4. Discussion

We found temporal variation patterns related to the weekend in the bat acoustic data but not in the weather data. Previous studies that reported the weekend effect on weather have been conducted either in major metropolitan areas (e.g., Melbourne, Australia, [17]; Guangzhou, China, [21]) or over large multi-city spatial scales (e.g., [18,20]). In our study, Greensboro is a medium-sized city that is much smaller than cities in previous studies in terms of both the absolute population size and the population density. It is not surprising that leisure activities on weekends might not release enough compounds into the atmosphere to trigger weather changes. Additionally, we defined Friday and Saturday nights as weekends to study nocturnal bats. The definition of the weekend might be different from meteorological studies cited above.

Without evident weather variations related to days of the week, the weekend effect found in bat acoustic activity is likely explainable by direct disturbances caused by human activities. Contradictory to our hypothesis or previous wildlife studies that reported unidirectional effects of the weekend (e.g., [25,26]), we found two different weekend effect patterns at two locations. At the UNCG sites, we observed a decrease of bat activity on weekends, which is consistent with the known negative weekend effect on wildlife. However, at the GSC sites, we observed an increase of bat activity on weekends, which has not been reported by any previous studies in any groups of wildlife. We suspect that different urban settings between locations might explain the contradictory weekend effect patterns.

Near the UNCG sites, there are many venues for leisure activities. Even though our study did not measure human activities quantitatively, it is evident that more patrons visited restaurants, bars, and sporting events in the area on weekend nights. Specifically, several outdoor sport facilities (within 1 km from the UNCG sites) usually schedule sporting events and firework shows on weekend nights during our study seasons, which can increase the light and noise level temporally and disturb bats (e.g., [41,44]). In addition to local residents, students on the university campus might be more active on weekend nights than on weekdays. In contrast, the GSC sites are located in a park complex that is closed to the public in the evening. The surrounding low-density residential area lack venues for nighttime leisure activities and generally does not attract any types of people gathering at night (Figure S1). The difference in urban settings can form different levels of human disturbance. Thus, we suspect that bats might fly away from the UNCG sites and disperse to the peripheral residential-dominated areas of the city when human disturbances increase on weekends in the downtown area.

In urban areas, bats have been known to select habitats actively and display directional movements in the habitat mosaic (e.g., [33,57,58]). Such movements are usually associated with roost rotations [59]. At the GSC sites, the hourly bat activity patterns showed a bat activity spike near sunrise on Friday night. We suspect that this spike might indicate bats searching for roosts before sunrise and these bats might only roost near the GSC sites on the weekends. In contrast with Friday, the hourly bat activity on Saturday was consistently higher, suggesting bats that arrived on Friday night might stay in the area. Previous studies have shown that bats switch roosts seasonally [60,61] or due to natural environmental changes [62,63]. Our work provides indirect evidence of bat movement and roost switching due to human disturbances. We recommend that future studies should employ radio transmitters or GPS tracking techniques to find direct evidence on how human disturbances affect urban bat movements.

Among seven species in our study area, we found the weekend effect in four of them. The species-specific patterns of the four species are generally consistent with the overall pattern of all bats combined. Additionally, the four species are also the more commonly recorded species. Thus, we suspect that all species in our study area might respond to human disturbances in a similar way as suggested by Moretto and Francis [64]. We did not find the weekend effect in the other three species. We suspect that it might be because of the low numbers of recordings and the consequential lack of statistical power to detect patterns [54]. Interestingly, at the GSC site where the park complex has more trees than buildings, we found the highest increases in the big brown bat and the evening bat. However, we mostly observe these bats roosting in buildings in our study area. Furthermore, the big brown bat has shown roost switching behaviors (switching locations and/or roost types, e.g., [60,61,65]) but the evening bat is known for the fidelity to certain roosts [65]. Perhaps bats in our study area were flexible in roost selection to cope with urban disturbances or buildings in the park complex were preferred by those bats [66]. We believe there is the need for studies on bat roost ecology.

We found the weekend effect mainly during summer. This is because during summer bats are more sedentary than spring or fall. In spring and fall, many bats are involved with long distance seasonal movement to/from overwinter grounds [67]. Our study area is on the migratory pathway for many species [68] and any fluctuation in bat acoustic activity might be related to migrating individuals passing through. We suggest that future research should consider other cities in different geographical regions or comparisons of multiple cities and broader spatial scales.

## 5. Conclusions

In the urban environment, human presence and human footprint on the environment are different concepts [69]. Our work demonstrates that weekend leisure activities might not cause the weather condition changes but the presence of humans can alter bat activity. Within a city, wildlife can adapt to temporally patterned human activities and balance the cost of movement and the benefit of avoiding disturbance [65]. From the ecological perspective, our work suggests the importance of behavioral adaptability for wildlife to cope with the urbanizing world. From the wildlife conservation and management as well as the public health perspective, such adaptability may increase wildlife movements in the city and potentially increase the chance of human-wildlife interaction and the risk of wildlife disease spillover. We recommend that urban planning should implement practices such as adding new greenspaces and/or preserving old-growth vegetation to form continuous greenways from the city center to the city periphery as corridors to facilitate bat movements. It is beneficial to provide a favorable habitat for bats for roosting and foraging in urban spaces to encourage bat species presence, which assists in naturally managing the nocturnal insect populations.

## Figures and Tables

**Table 1:** Numbers of total bat passes (including no species identification) collected in 2018 and 2019 springs, summers, falls and generalized linear model results (total bat passes versus day of the week and environmental covariates, regression estimate ± standard error and *p* value reported) by season at the Greensboro Science Center sites in Greensboro, North Carolina, USA. “NA” indicates variables dropped from the final model. Bold numbers indicate significant results.

Variable	Greensboro Science Center Open					
	Spring		Summer		Fall	
Bat Passes	6131		20,490		20,857	
Day–Tuesday	0.377 ± 0.248	0.132	0.006 ± 0.326	0.984	0.378 ± 0.313	0.231
Day–Wednesday	0.185 ± 0.242	0.446	−0.102 ± 0.345	0.769	0.489 ± 0.288	0.094
Day–Thursday	0.324 ± 0.237	0.175	0.063 ± 0.338	0.852	−0.397 ± 0.329	0.231
Day–Friday	**0.611 ± 0.230**	**0.010**	**0.763 ± 0.293**	**0.011**	−0.627 ± 0.385	0.084
Day–Saturday	**0.496 ± 0.233**	**0.036**	**0.723 ± 0.292**	**0.015**	−0.003 ± 0.335	0.994
Day–Sunday	0.316 ± 0.239	0.190	0.245 ± 0.315	0.439	−0.416 ± 0.366	0.260
Temperature (°C)	**0.170 ± 0.014**	**<0.001**	**0.093 ± 0.039**	**0.020**	**0.240 ± 0.024**	**<0.001**
Wind (km/h)	NA		**−0.185 ± 0.075**	**<0.001**	NA	
Year	**−0.435 ± 0.155**	**0.006**	NA		NA	
	Greensboro Science Center Woods					
Bat Passes	2782		8643		2099	
Day–Tuesday	0.269 ± 0.328	0.415	−0.118 ± 0.278	0.673	0.034 ± 0.239	0.886
Day–Wednesday	0.247 ± 0.331	0.459	−0.258 ± 0.291	0.378	−0.277 ± 0.256	0.283
Day–Thursday	0.394 ± 0.388	0.201	−0.071 ± 0.283	0.802	−0.319 ± 0.252	0.209
Day–Friday	0.061 ± 0.316	0.849	**0.929 ± 0.230**	**<0.001**	−0.346 ± 0.264	0.193
Day–Saturday	−0.074 ± 0.333	0.825	**0.869 ± 0.231**	**<0.001**	−0.015 ± 0.254	0.953
Day–Sunday	0.409 ± 0.311	0.194	0.275 ± 0.258	0.290	0.090 ± 0.246	0.716
Temperature (°C)	**0.130 ± 0.016**	**<0.001**	**0.181 ± 0.027**	**<0.001**	**0.122 ± 0.013**	**<0.001**
Wind (km/h)	NA		**−0.078 ± 0.034**	**0.047**	NA	
Year	−1.258 ± 0.165	**<0.001**	NA		NA	

**Table 2:** Numbers of total bat passes (including no species identification) collected in 2018 and 2019 springs, summers, falls and generalized linear model results (total bat passes versus day of the week and environmental covariates, regression estimate ± standard error and p value reported) by season at the University of North Carolina Greensboro sites in Greensboro, North Carolina, USA. “NA” indicates variables dropped from the final model. Bold numbers indicate significant results.

Variable	University of North Carolina Greensboro Open					
	Spring		Summer		Fall	
Bat Passes	44,532		49,139		12,814	
Day–Tuesday	−0.178 ± 0.230	0.438	0.053 ± 0.231	0.820	−0.367 ± 0.393	0.340
Day–Wednesday	0.007 ± 0.206	0.973	0.009 ± 0.241	0.970	−0.030 ± 0.358	0.933
Day–Thursday	0.072 ± 0.197	0.714	−0.219 ± 0.246	0.074	0.078 ± 0.346	0.822
Day–Friday	**−0.321 ± 0.218**	**0.043**	**−0.524 ± 0.257**	0.016	−0.145 ± 0.369	0.695
Day–Saturday	**−0.660 ± 0.217**	**0.016**	**−0.264 ± 0.232**	**0.020**	0.091 ± 0.356	0.799
Day–Sunday	−0.305 ± 0.233	0.089	0.012 ± 0.239	0.958	0.037 ± 0.369	0.920
Temperature (°C)	**0.115 ± 0.011**	**<0.001**	**0.078 ± 0.030**	**0.010**	**0.097 ± 0.019**	**<0.001**
Wind (km/h)	NA		**−0.035 ± 0.019**	**0.017**	**−0.029 ± 0.007**	**0.044**
Year	NA		**0.513 ± 0.138**	**<0.001**	**−0.439 ± 0.203**	**0.033**
	University of North Carolina Greensboro Woods					
Bat Passes	9185		1057		2985	
Day–Tuesday	0.141 ± 0.460	0.759	0.243 ± 0.339	0.474	0.055 ± 0.489	0.911
Day–Wednesday	0.258 ± 0.436	0.554	0.196 ± 0.353	0.579	−0.605 ± 0.569	0.290
Day–Thursday	0.287 ± 0.433	0.508	−0.279 ± 0.385	0.471	−1.010 ± 0.655	0.126
Day–Friday	0.304 ± 0.440	0.491	−0.039 ± 0.383	0.918	−0.651 ± 0.578	0.263
Day–Saturday	−0.113 ± 0.483	0.815	0.167 ± 0.348	0.633	0.144 ± 0.474	0.763
Day–Sunday	−0.206 ± 0.500	0.681	0.105 ± 0.351	0.765	−0.283 ± 0.565	0.618
Temperature (°C)	**0.079 ± 0.020**	**<0.001**	**0.140 ± 0.042**	**0.001**	**0.067 ± 0.028**	**0.018**
Wind (km/h)	NA		NA		NA	
Year	NA		**0.530 ± 0.191**	**0.007**	**−0.595 ± 0.322**	**0.067**

**Table 3:** Numbers of species-specific bat passes collected in 2018 and 2019 summers and generalized linear model results (species-specific bat passes versus day of the week and environmental covariates, regression estimate ± standard error and p value reported) at the Greensboro Science Center open site in Greensboro, North Carolina, USA. “NA” indicates variables dropped from the final model. Bold numbers indicate significant results.

Variable	Big Brown Bat		Red Bat		Hoary Bat		Silver-Haired Bat	
Bat Passes	1449		1130		965		2471	
Day–Tuesday	0.175 ± 0.308	0.571	0.080 ± 0.173	0.719	−0.008 ± 0.271	0.976	−0.070 ± 0.384	0.463
Day–Wednesday	0.050 ± 0.327	0.878	0.156 ± 0.215	0.487	−0.196 ± 0.265	0.461	−0.156 ± 0.328	0.290
Day–Thursday	0.068 ± 0.332	0.838	−0.100 ± 0.188	0.688	−0.082 ± 0.291	0.778	−0.317 ± 0.335	0.320
Day–Friday	0.130 ± 0.332	0.067	0.013 ± 0.159	0.955	0.028 ± 0.284	0.922	**0.701 ± 0.369**	**0.005**
Day–Saturday	**0.547 ± 0.282**	**0.006**	**0.210 ± 0.165**	**0.030**	0.136 ± 0.269	0.614	**0.548 ± 0.320**	**0.029**
Day–Sunday	0.051 ± 0.326	0.876	**0.227 ± 0.169**	**0.025**	0.176 ± 0.263	0.505	−0.008 ± 0.290	0.987
Temperature (°C)	**0.116 ± 0.040**	**0.006**	**0.286 ± 0.066**	**0.031**	NA		NA	
Wind (km/h)	**−0.110 ± 0.047**	**0.005**	**−0.102 ± 0.032**	**0.002**	NA		**−0.330 ± 0.068**	**<0.001**
Year	**−0.483 ± 0.193**	**0.018**	NA		NA		NA	
	Evening Bat		Tricolored Bat		Mexican Free-Tailed Bat			
Bat passes	1993		285		799			
Day–Tuesday	0.003 ± 0.288	0.992	−0.548 ± 0.388	0.092	−0.329 ± 0.394	0.249		
Day–Wednesday	−0.029 ± 0.301	0.932	0.307 ± 0.422	0.501	−0.432 ± 0.433	0.230		
Day–Thursday	0.148 ± 0.271	0.149	0.293 ± 0.429	0.526	−0.485 ± 0.370	0.196		
Day–Friday	**0.478 ± 0.249**	**0.025**	0.146 ± 0.436	0.757	0.193 ± 0.371	0.088		
Day–Saturday	0.052 ± 0.300	0.881	0.361 ± 0.408	0.515	0.005 ± 0.297	0.987		
Day–Sunday	0.006 ± 0.296	0.986	0.078 ± 0.438	0.870	−0.173 ± 0.313	0.595		
Temperature (°C)	**0.123 ± 0.038**	**0.005**	NA		NA			
Wind (km/h)	**−0.187 ± 0.048**	**<0.001**	**−0.177 ± 0.066**	**0.008**	**−0.152 ± 0.052**	**0.004**		
Year	**−0.872 ± 0.209**	**<0.001**	**−1.097 ± 0.297**	**0.001**	NA			

**Table 4:** Numbers of species-specific bat passes collected in 2018 and 2019 summers and generalized linear model results (species-specific bat passes versus day of the week and environmental covariates, regression estimate ± standard error and *p* value reported) at the University of North Carolina Greensboro open site in Greensboro, North Carolina, USA. “NA” indicates variables dropped from the final model. Bold numbers indicate significant results.

Variable	Big Brown Bat		Red Bat		Hoary Bat		Silver-Haired Bat	
Bat Passes	28,996		757		151		533	
Day–Tuesday	0.025 ± 0.271	0.928	0.106 ± 0.283	0.712	0.610 ± 0.930	0.513	0.300 ± 0.359	0.404
Day–Wednesday	0.026 ± 0.283	0.929	0.095 ± 0.224	0.749	−0.317 ± 0.710	0.787	0.351 ± 0.359	0.330
Day–Thursday	−0.111 ± 0.281	0.066	−0.172 ± 0.292	0.279	−0.361 ± 0.772	0.756	0.127 ± 0.371	0.733
Day–Friday	**−0.496 ± 0.294**	**0.015**	**−0.465 ± 0.233**	**0.028**	0.556 ± 0.953	0.560	0.150 ± 0.377	0.692
Day–Saturday	**−0.355 ± 0.206**	**0.022**	**−0.349 ± 0.189**	**0.039**	0.917 ± 0.898	0.309	0.175 ± 0.377	0.643
Day–Sunday	0.090 ± 0.272	0.746	0.133 ± 0.252	0.649	1.428 ± 0.850	0.095	0.164 ± 0.381	0.666
Temperature (°C)	**0.079 ± 0.031**	**0.017**	**0.195 ± 0.037**	**<0.001**	NA		NA	
Wind (km/h)	**−0.082 ± 0.037**	**0.029**	**−0.082 ± 0.043**	**0.050**	NA		NA	
Year	**0.711 ± 0.160**	**<0.001**	**0.777 ± 0.170**	**<0.001**	NA		**−0.598 ± 0.198**	**0.003**
	Evening Bat		Tricolored Bat		Mexican Free-Tailed Bat			
Bat Passes	1943		268		243			
Day–Tuesday	0.251 ± 0.390	0.191	0.800 ± 0.467	0.070	0.767 ± 0.431	0.074		
Day–Wednesday	0.193 ± 0.329	0.654	0.329 ± 0.539	0.520	0.332 ± 0.465	0.472		
Day–Thursday	0.072 ± 0.373	0.867	0.335 ± 0.456	0.500	0.332 ± 0.446	0.467		
Day–Friday	**−0.525 ± 0.362**	**0.037**	0.404 ± 0.500	0.420	0.539 ± 0.442	0.230		
Day–Saturday	**−0.664 ± 0.384**	**0.023**	0.469 ± 0.450	0.350	0.736 ± 0.456	0.097		
Day–Sunday	0.205 ± 0.400	0.208	0.551 ± 0.475	0.250	0.511 ± 0.441	0.261		
Temperature (°C)	**0.085 ± 0.044**	**0.006**	**0.260 ± 0.058**	**<0.001**	**0.106 ± 0.046**	**0.021**		
Wind (km/h)	NA		**−0.168 ± 0.069**	**0.016**	NA			
Year	**1.526 ± 0.274**	**<0.001**	NA		**−0.978 ± 0.241**	**<0.001**		

## Supplementary Materials

The following are available online at https://www.mdpi.com/2076-2615/10/9/1636/s1, Figure S1: Maps and aerial photos of study area and sites, Figure S2: Photo demonstration of the bat detector setup at the Greensboro Science Center open site, Table S3: Generalized linear models for weather and bat data and model results for weather data, in which no significant result was found.

## Appendix A

All bat data are deposited to BatAMP (https://batamp.databasin.org/) and NABat National Database (https://www.nabatmonitoring.org/). Weather data were downloaded from publicly available weatherSTEM database for UNCG (https://guilford.weatherstem.com/data?refer=/uncg). No novel R code was developed for this study. All R codes for statistical analysis and data visualization are available from the corresponding author on reasonable requests.
