# Supplementary figures and images for: The Weekend Effect on Urban Bat Activity Suggests Fine Scale Human-Induced Bat Movements
Source: Animals (Basel). 2020 Sep 11;10(9):1636. doi: 10.3390/ani10091636 (PMC7552248; doi:10.3390/ani10091636)

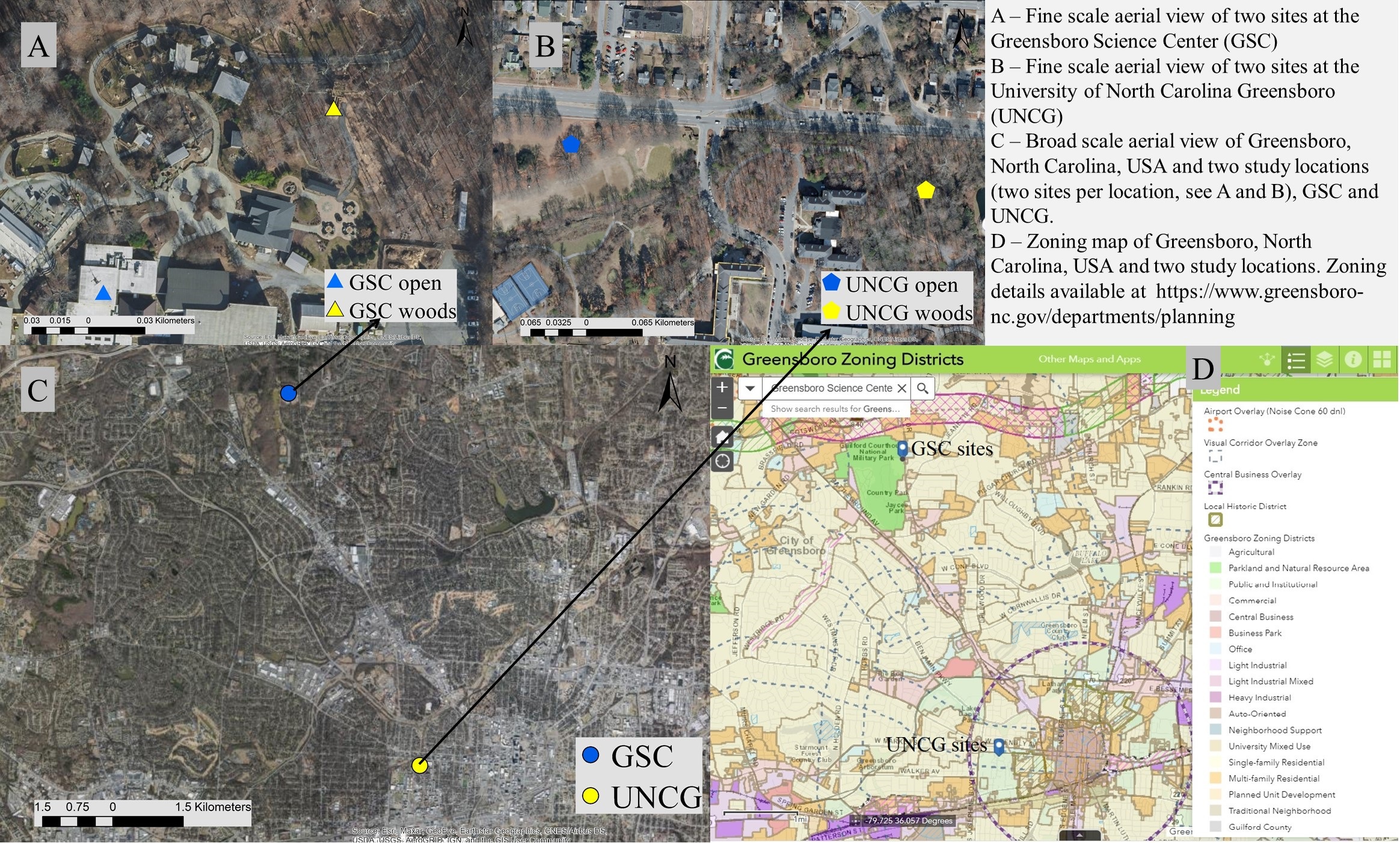

Supplement: Supplementary file 1 [file animals-10-01636-s001.zip › Supplemetary files-R1/Figure S1.jpg]

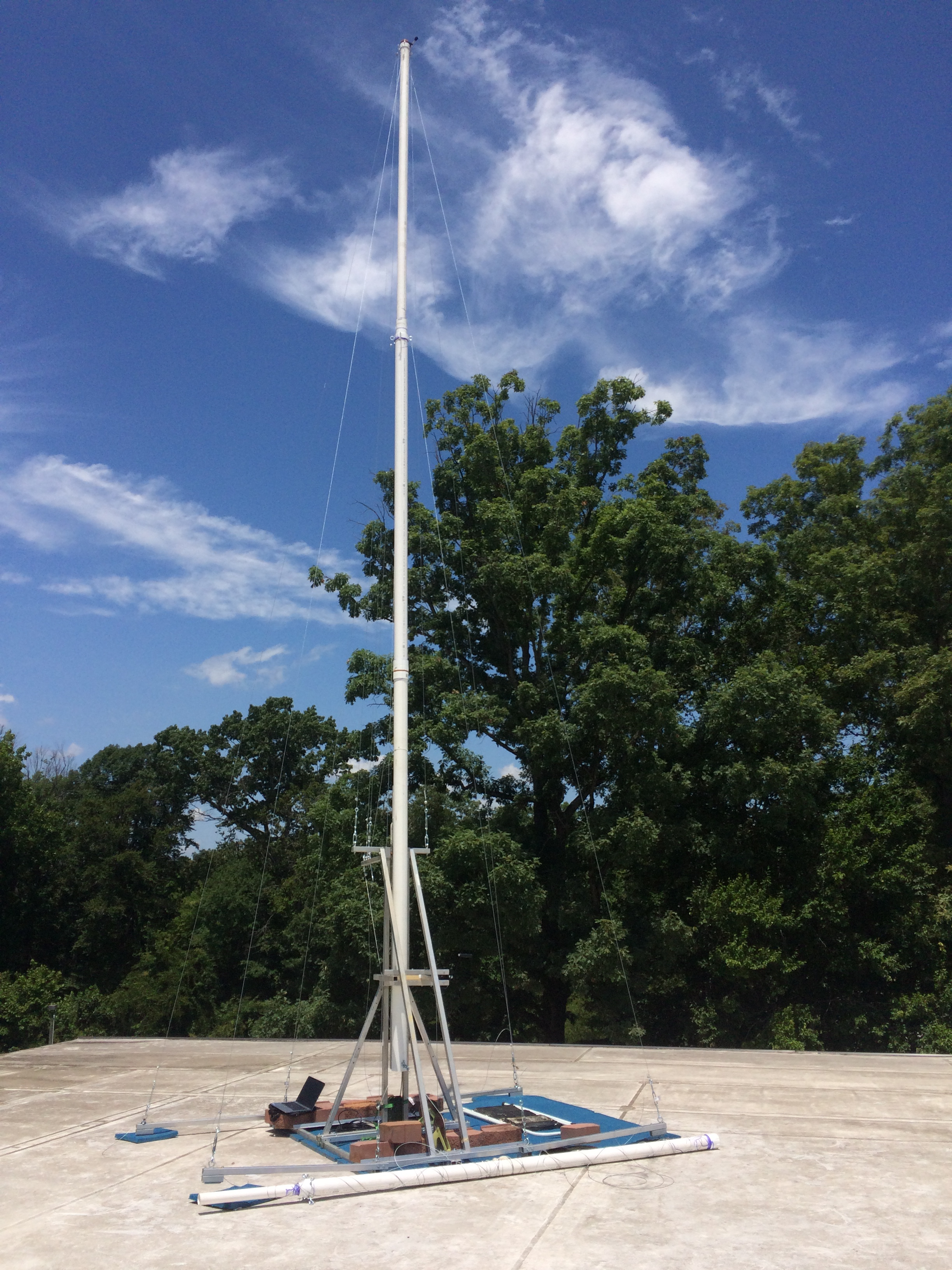

Supplement: Supplementary file 1 [file animals-10-01636-s001.zip › Supplemetary files-R1/Figure S2.jpg]
